# Surgical Robots Improve Tunnel Angle and Graft Bending Angle in Anatomic ACL Reconstruction: A Multicenter Study

**DOI:** 10.3390/bioengineering12040338

**Published:** 2025-03-24

**Authors:** Ling Zhang, Hansheng Hu, Wennuo Huang, Mengling Hu, Zhuman Li, Jinzhong Zhao, Wenyong Fei, Shaobai Wang

**Affiliations:** 1 School of Exercise and Health, Shanghai University of Sport, 200 Hengren Road, Shanghai 200438, China; 2 Department of Orthopedics and Sports Medicine, Northern Jiangsu People’s Hospital Affiliated to Yangzhou University, 98 Nantong West Road, Yangzhou 225001, China; 3 Department of Radiological Sciences, Northern Jiangsu People’s Hospital Affiliated to Yangzhou University, Yangzhou 225001, China; 4 Department of Sports Medicine, Shanghai Sixth People’s Hospital, Shanghai Jiao Tong University, Shanghai 200233, China

**Keywords:** knee, robot, anatomical reconstruction, tunnel angle, graft bending angle

## Abstract

The anatomic characteristics of the graft and tunnel, i.e., the tunnel position, angle, length, and the graft bending angle, influence knee joint stability and postoperative functional recovery. The purpose of this study was to evaluate the tunnel position, length and angle, as well as graft bending angle after ACL reconstruction assisted by a surgical robot. A total of 70 patients were randomized into two groups: the surgical robot group (robot group, *n* = 35) and the traditional handheld locator group (control group, *n* = 35). Postoperative computed tomography (CT) was employed to assess the positions and lengths of the tunnels, as well as the tunnel angle and the graft bending angle. Additionally, the posterior wall distance was measured by determining the minimum vertical distance from the long axis of the tunnel to the posterior wall region. There were no significant differences between the two groups in the mean position or length of the femoral and tibial tunnel (*p* > 0.05). However, the femoral tunnel angle was significantly larger in the robot group compared to the handheld locator group (*p* = 0.012). The graft bending angle was significantly less acute in the robot group than in the control group (*p* = 0.008). Additionally, the posterior wall distance was significantly greater in the robot group compared to the control group (*p* < 0.001). The results suggest that surgical robot-assisted ACL reconstruction enhances safety in the inclination of the tunnel and graft, helping to avoid potential biomechanical issues such as the wiper effect and the bungee effect, which may lead to tunnel widening and surgical failure.

## 1. Introduction

Recent advances in anterior cruciate ligament (ACL) reconstruction have emphasized anatomical reconstruction techniques aimed at more effectively restoring the native structure and function of the knee joint [1,2]. Despite these surgical improvements, studies indicate that approximately 10~40% of bone tunnels in primary ACL reconstructions are inaccurately placed, which is strongly associated with clinical failure after the procedure [3,4,5]. Postoperative imaging studies have revealed inter-surgeon variability ranging from 19% to 34% in tunnel placement when using only arthroscopic guidance [6,7]. This may be attributed to the inherent challenges of assessing relative depth and perspective with a monocular arthroscope, as well as the difficulty in accurately identifying intra-articular landmarks [8].

Despite two decades of development in computer navigation systems for planning, identifying, and drilling accurate ACL reconstruction bone tunnels [9,10,11,12,13], these systems still rely on manual surgical execution where human error remains unavoidable [11,14,15]. In contrast, surgical robots provide a more precise approach by drilling tunnels based on pre-determined paths and angles [11]. This automation reduces the effects of hand tremors and improves surgical accuracy and consistency. Ding et al. [11] conducted a cadaveric study comparing the precision of tunnel creation for ACL reconstruction using the TiRobot surgical robot to a traditional handheld locator. Their results showed that the TiRobot achieved a significantly higher level of accuracy, with measurements of 1.00 mm (±0.20 mm) for the TiRobot compared to 3.10 mm (±0.59 mm) for the traditional method [11]. Additionally, Yang et al. [10] demonstrated through a cadaveric study that surgical robots utilized in ACL reconstruction can create femoral tunnels that align more closely with the anatomical ACL footprint. While robot-assisted ACL reconstruction has shown promise in cadaver studies [3,9,10,16], clinical validation through real-world surgical applications remains notably absent.

In recent years, researchers have emphasized that the primary goal of anatomical ACL reconstruction is not only to achieve an anatomically accurate tunnel position but also to restore anatomical tunnel and graft orientation [17,18]. To achieve more anatomically accurate ACL reconstruction, modifications to the traditional transtibial technique have been proposed [19]. However, these advancements require a higher level of clinical precision and expertise [19]. Furthermore, anatomical ACL reconstruction methods involve inherent risks, such as a short femoral tunnel, posterior wall blowout, and potential iatrogenic damage to the medial femoral condyle [14,20]. These risks are primarily associated with the more horizontal orientation of the femoral tunnel in three-dimensional space [20,21]. Despite these advancements, clinical research evaluating the precision of surgical robots in ACL reconstruction and their impact on the anatomical characteristics of tunnels and grafts remains limited and inconclusive.

The purpose of this study was to assess the tunnel position, length and angle, graft bending angle, and distance to the posterior wall following anatomical ACL reconstruction by comparing the use of a surgical robot with a traditional handheld locator. It was hypothesized that surgical robot-assisted ACL reconstruction would result in a larger femoral tunnel angle, a less acute graft bending angle, and an increased distance to the posterior wall compared to traditional handheld locator techniques.

## 2. Materials and Methods

### 2.1. Study Approvals

The study protocol was approved by the hospital review board (2022058). Written informed consent was obtained from all participating patients before they were randomized.

### 2.2. Study Population

The study included skeletally mature patients aged 18 to 60 who required unilateral ACL reconstruction, with or without a partial meniscectomy or meniscal repair. Patients with a history of previous knee surgery, revision surgery, multiligamentous injuries, or degenerative joint disease on the same side were excluded from this study. Basic demographic information was collected for each patient.

### 2.3. Group Allocation

**Figure 1:**
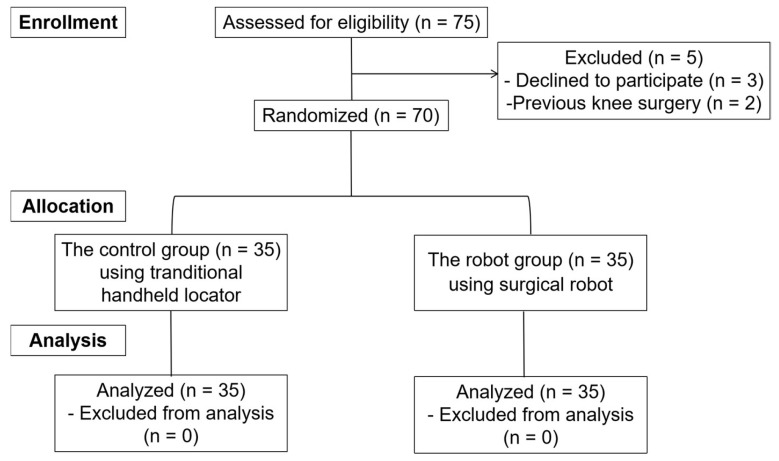
Consolidated Standards of Reporting Trials (CONSORT) flow diagram of the study.

From April 2022 to November 2023, a total of 70 patients were enrolled in this study, as shown in the patient flow chart (Figure 1). Upon obtaining consent, patients were randomly assigned using a random number generator to either the robot group (robot group) or the traditional handheld locator group (control group), with 35 patients allocated to each group. There were no significant differences between the groups regarding age, sex, BMI, time from injury to surgery, and meniscal surgery (Table 1). Patients were unblinded to their assigned surgical method, while the investigators conducting the statistical analysis were blinded to group assignments. After completing the analysis, the data were unblinded for the final interpretation of the results.

### 2.4. Surgical Robot Procedures

**Figure 2:**
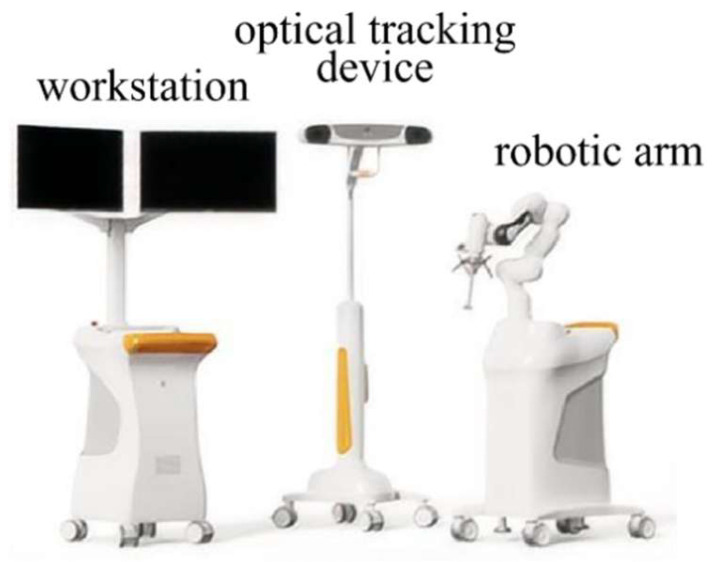
Composition of Intelligent Knee Stability Restoration Surgical Robot System.

Robot preparation: The Intelligent Knee Stability Restoration (IKSR) surgical robot system (DroidSurg Medical Co., Ltd., Shanghai, China) was utilized for anatomical single bundle ACL reconstruction in combination with an arthroscopic system. This system consists of a workstation, an optical tracking device, and a robotic arm (Figure 2) that facilitates ACL insertion point registration, tunnel planning, and drilling. The surgical robot is equipped with two specially designed femoral and tibial locators, as well as femoral and tibial trackers. The workstation allows for planning and adjusting bone tunnel positions. The optical tracking device provides real-time tracking of the spatial positions of the patient’s knee. And the robotic arm, featuring 7 degrees of freedom, can navigate and position itself flexibly to accurately reach the planned surgical site. During the surgical procedure, the robotic arm is positioned on the same side as the surgeon, while the workstation and optical tracking device are placed on the opposite side to avoid obstruction.

**Figure 3:**
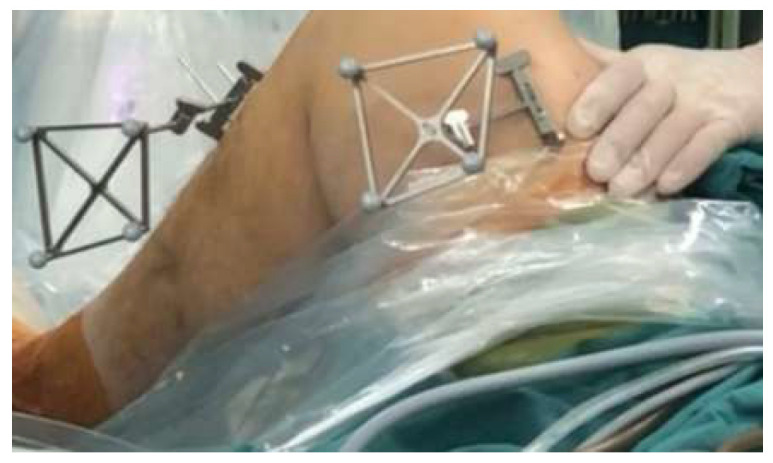
Fixation of femoral and tibial trackers.

**Figure 4:**
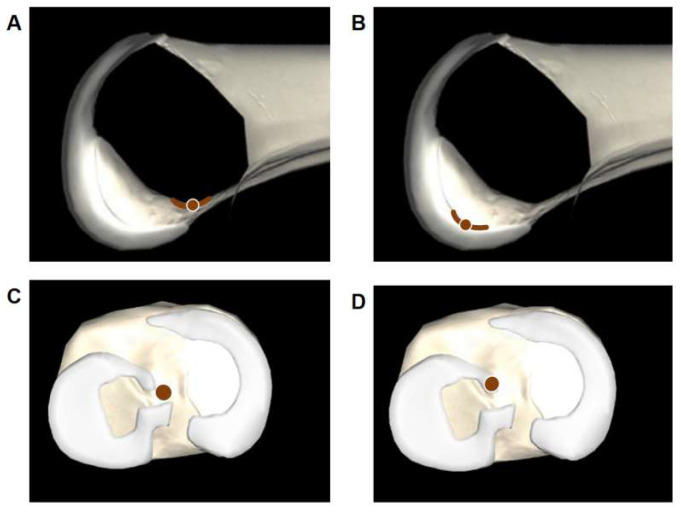
Identification of intra-articular reference points using a locator: the intercondylar notch roof line (**A**), the lateral notch wall (**B**), the medial intercondylar tubercle (**C**), and the anterior horn of the lateral meniscus (**D**).

Intraoperative planning: The trackers were first fixed to the lateral femur and medial tibia (Figure 3). The knee was passively extended under controlled conditions, with a 5 kg sandbag placed above it to ensure full anatomical extension. The lower limb was positioned flat on the table, allowing that the weight effectively contributed to fully extending the knee throughout the process. Following this, the centers of the hip and knee joints were identified according to the step-by-step instructions displayed on the workstation. Using a locator, the surgeon marked the medial and lateral points of the ankle and knee joints to define relative spatial relationships of the lower limb. Subsequently, a specially designed femoral locator was employed to identify inter-articular reference points, including the medial notch wall, the intercondylar notch roof line, and the lateral notch wall (Figure 4A,B). Similarly, the medial intercondylar tubercle on the tibial plateau and the anterior horn of the lateral meniscus were identified using the tibial locator (Figure 4C,D). These reference points, as proposed by Zhao [19,22], were used to determine the anatomical positions for the femoral and tibial tunnels. The locator position can be displayed on the workstation in real time during tunnel planning. Once inter-articular reference points were registered, the system automatically generated the preoperative planned bone tunnels.

Intraoperative navigated drilling: Bone tunnels were drilled according to the planned pathways using the robotic arm. It was crucial to maintain the trackers and the end of the robotic arm within the field of view of the optical tracking device. This ensured that the workstation could continuously monitor and acquire real-time spatial positions of both the knee and the robotic arm. The robotic arm navigated according to the predetermined trajectory, with its position and any deviations from the planned tunnel being displayed in real time on the workstation. A 2.4 mm Kirschner wire was inserted from the tibia into the femur by the surgeon through the guide sheath at the end of the robotic arm. And the ACL femoral and tibial tunnels were drilled along the Kirschner wire using an 8 mm drill.

### 2.5. Traditional Arthroscopic Procedures

With the patient positioned supine, an anterolateral portal was established for arthroscopic examination to assess any related injuries. Partial or subtotal meniscectomy, as well as meniscal repair, was performed for tears of the lateral and/or medial meniscus. The semitendinosus and gracilis tendons were harvested, folded, and sutured to create a four-strand graft. During the surgical procedure, ACL remnants were debrided to enhance visualization during arthroscopy. Key bony landmarks, including the lateral intercondylar and bifurcate ridge, were identified to determine anatomical femoral tunnel position. Intra-articular reference points, such as the inner edge of the anterior horn of the lateral meniscus and the intercondylar eminence of the tibial plateau, were used as reference points for tibial tunnel placement. Subsequently, Kirschner wires and bone tunnel drills with an 8 mm diameter were employed to create the bone tunnels. The passage and fixation of the ACL graft were performed according to standard protocols, using interference screw fixation on the tibia and cortical button fixation on the femur.

### 2.6. Postoperative Measurement

**Figure 5:**
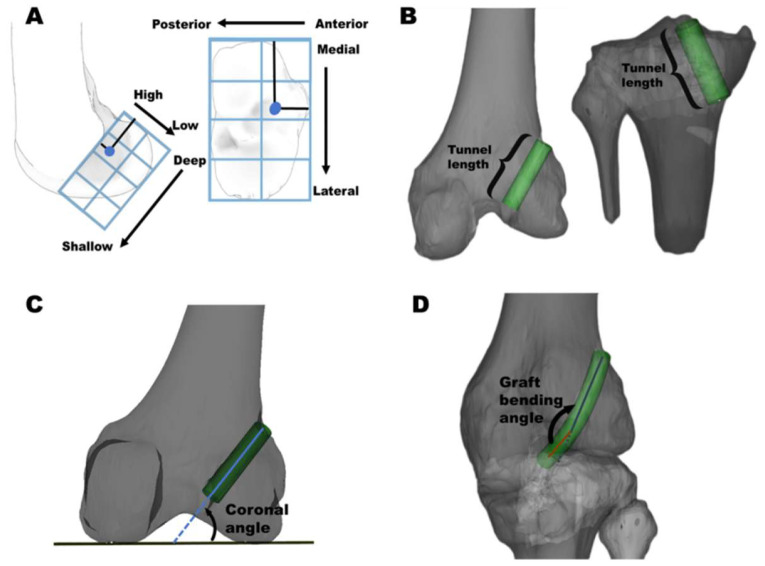
(**A**) Femoral and tibial tunnel positions: femoral tunnel position was quantified as a percentage relative to the dimensions of the lateral femoral condyle, while tibial tunnel position was expressed as a percentage concerning the tibial plateau in the medial-lateral and anterior-posterior directions. (**B**) Tunnel length: distance from the inner to the outer apertures of the tunnel. (**C**) Femoral coronal angle: angle formed between the tunnel and a line tangent to the distal femoral joint line. (**D**) Graft bending angle: angle between the femoral tunnel and the graft.

**Figure 6:**
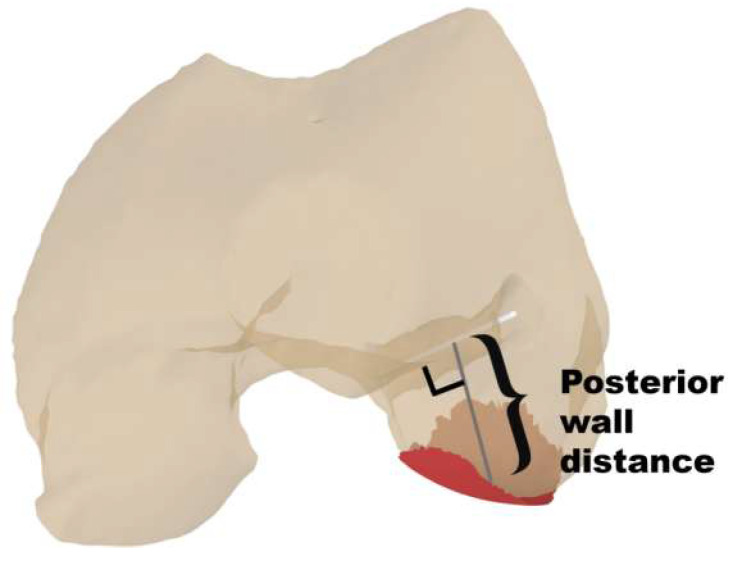
Calculation of posterior wall distance: minimum vertical distance from the long axis of the femoral tunnel to the posterior wall region.

To evaluate femoral and tibial tunnel positions, 3D CT imaging was performed with the knee maintained in full extension at one-week post-surgery. The tunnel location was determined by the center point at the inner aperture of both the femoral and tibial tunnels using the quadrant method as described by Bernard et al. [23] (Figure 5A). Secondary outcomes included tunnel lengths, coronal angles, graft bending angles, and distances to the posterior wall region (Figure 5 and Figure 6).

Additionally, the posterior wall distance is calculated by identifying the minimum vertical distance from the long axis of the tunnel to all points within the posterior wall region. This is achieved through a detailed analysis of the three-dimensional (3D) femoral model. Specifically, the tunnel’s long axis is defined, and the posterior wall region is segmented within the 3D model. Using computational algorithms, all points along the posterior wall are assessed, and the shortest perpendicular distance from the tunnel axis to these posterior wall points is determined. This measurement ensures precise assessment of the proximity of the tunnel to the posterior cortex, which is critical for evaluating potential risks of posterior wall blowout.

### 2.7. Statistical Analysis and Sample Size Calculation

Based on findings from a prior cadaveric study [9], the mean deviation between the actual and pre-planned femoral tunnel positions was 1.8 ± 0.4 mm in the robot-assisted group and 3.4 ± 1.9 mm in the traditional handheld locator group. A difference (delta) of 1.6 mm was considered clinically meaningful [24], as it reflects a potentially significant impact on femoral tunnel placement and subsequent knee stability. The power analysis was based on the larger standard deviation of the traditional handheld locator group (3.4 ± 1.9 mm) to ensure that sample size calculation reflects the most conservative estimate. Using PASS 16.0 software, the sample size was calculated with a power of 90%, an alpha level of 0.025, and the observed standard deviations. The calculation indicated that 19 participants per group were required. To account for an anticipated dropout rate of 10%, the final sample size was set at 22 participants per group.

An independent *t*-test was employed for comparing groups with normally distributed variables, while the Mann–Whitney U test was utilized for data that did not follow a normal distribution. For the analysis of categorical data, the Pearson chi-square test was employed. Statistical analyses were conducted using SPSS (v 26; IBMCorp., Armonk, NY, USA), with a significance level set at *p* < 0.05.

## 3. Results

### 3.1. Tunnel Position

**Figure 7:**
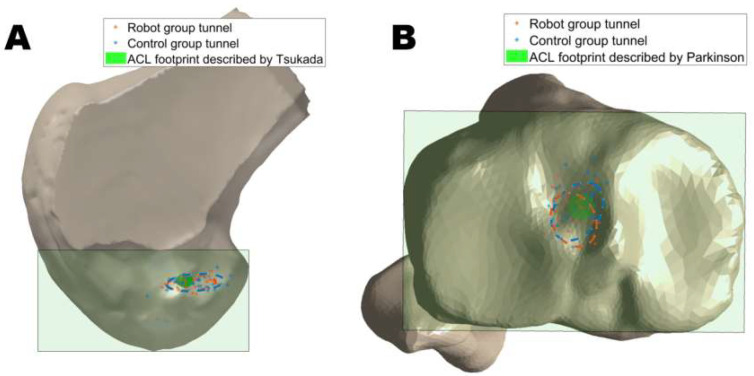
Distribution of femoral tunnel locations (**A**) and tibial tunnel locations (**B**) from robot and control groups. * represents tunnel location for each case. Dashed ellipses represent 95% confidence distributions of tunnel locations in both robot and control groups. Green shaded areas represent the central area of ACL footprints reported by Tsukada et al. [25] and Parkinson et al. [26].

There was no significant difference between the two groups in the mean position of the femoral tunnel center in either the deep-shallow direction (*p* = 0.315) or the high-low direction (*p* = 0.700). Similarly, no significant difference was observed in the mean position of the tibial tunnel center in the anteroposterior direction (*p* = 0.110) or the mediolateral direction (*p* = 0.247). The distribution of the tunnel position is shown in Figure 7.

### 3.2. Tunnel Length

The mean femoral tunnel length was shorter in the control group compared to the robot group, but the difference was not statistically significant (34.4 ± 5.4 mm vs. 35.6 ± 5.4 mm, *p* = 0.386). Similarly, the mean tibial tunnel length in the control group was shorter than in the robot group, with no significant difference (31.5 ± 6.4 mm vs. 34.1 ± 7.5 mm, *p* = 0.173).

### 3.3. Tunnel and Graft Obliquity

In the coronal orientation, the femoral tunnel angle was significantly larger in the robot group (51.7° ± 7.2°) compared to the traditional handheld locator group (46.8° ± 8.5°, *p* = 0.012). The graft bending angle was significantly higher in the robot group (121.1° ± 11.7°) than in the control group (113.4° ± 13.1°, *p* = 0.008). Additionally, the posterior wall distance was significantly greater in the robot group (13.2 mm ± 2.9 mm) compared to the control group (10.3 mm ± 3.5 mm, *p* < 0.001), as summarized in Table 2.

## 4. Discussion

The key finding of this study was that robot assisted ACL reconstruction can achieve a less acute graft bending angle and a greater posterior wall distance. Additionally, surgical robots can achieve tunnel positions aligned with expert physicians with no significant differences in the mean positions or lengths of femoral and tibial tunnels. These results suggest that robot-assisted ACL reconstruction helps to avoid potential biomechanical problems, such as the ‘wiper effect’ and the ‘bungee effect’, which may contribute to surgical failure.

Accurate anatomical placement of the femoral tunnel is essential for optimizing knee function, enhancing joint stability, and reducing the risk of surgical failure [27,28]. Our analysis demonstrated that the surgical robot achieves alignment with anatomical ACL insertion points comparable to traditional handheld locator methods. However, as shown in the distribution of the femoral and tibial tunnels, the tunnel positions in the robot group were more centralized compared to the control group. It indicated that the positioning of the bone tunnels in robotic surgery exhibits less variability and aligns more closely with the anatomical ACL insertion points (Figure 7). This may be related to the experience of the different surgeons in the control group, whereas the accuracy of the surgical robot in drilling bone tunnels is less dependent on the individual surgeon’s experience, resulting in more consistent results across different specialists [29]. The surgical robot system used in the present study generates a 3D knee joint model by capturing intra-articular landmarks and surfaces, thereby eliminating the need for preoperative imaging and relying instead on real-time data collected during the procedure [9]. By employing kinematic analysis and identifying specific landmarks with an infrared locator, software algorithms can calculate and recommend optimal anatomical tunnel positions [9]. Furthermore, surgical robots could operate based on pre-set drilling paths and angles, significantly minimizing errors often associated with human factors, such as hand tremors, thus enhancing overall surgical accuracy and consistency.

However, the implementation of robot-assisted surgery often involves an initial increase in the duration of procedures [11]. This is attributed to several factors, including preoperative planning and the set-up and calibration of the robotic system [11]. As surgeons gain experience with robotic systems, the duration of procedures can decrease as surgeons become accustomed to the technology [30]. Additionally, the initial increase in procedure time may be offset by long-term benefits, such as reduced complication rates, improved outcomes, and shorter recovery times for patients [30]. The effective use of robotic-assisted techniques requires specialized training for surgeons. Managing these advanced tools and adapting to robotic methods can be challenging initially, which may slow technology adoption. However, once surgeons gain experience, the potential benefits, such as improved precision and outcomes, can be significant.

The study results also showed that the tibial and femoral tunnels created by the robot group were longer than those created by the traditional handheld locator group, though the difference was not statistically significant. Various advanced biomechanical methods are increasingly being used in the medical field to assess postoperative orthopedic outcomes [31,32]. Studies have shown that femoral tunnels shorter than 25 mm and tibial tunnels shorter than 30 mm reduce the effective healing length between the tendon and bone, ultimately weakening healing strength [33,34]. The femoral tunnel lengths in the robot and control groups measured 36 mm and 34 mm, respectively, indicating that both were sufficiently adequate. Similarly, the tibial tunnel lengths in the robot and control groups measured 34 mm and 31 mm, respectively. Trofa et al. [35] conducted a comparative analysis using postoperative 3D-CT scans and found that the anatomical ACL reconstruction had a significantly higher proportion of femoral tunnels shorter than 30 mm compared to the non-anatomical reconstruction. Consequently, robot-assisted ACL reconstruction not only facilitates anatomical ACL reconstruction, but also minimizes the risk of creating overly short tunnels.

The success of ACL reconstruction not only hinges on the accuracy of tunnel placement but also on the tunnel and graft obliquity [36]. In this study, the coronal plane angle of the femoral tunnel was significantly higher in the robot group compared to the control group, indicating that the femoral tunnel created with the traditional handheld locator was relatively more horizontal. Although a tunnel placed too horizontally may compromise rotational stability and risk lateral cortex proximity, an excessively steep (i.e., more vertical) femoral tunnel can likewise increase graft tension and predispose to PCL impingement [37]. Furthermore, Tachibana et al. [38] reported that as the coronal plane angle increases, the rate of postoperative tunnel enlargement can exceed 60%, suggesting that overly vertical tunnels also have disadvantages. Therefore, while robotic assistance enables real-time adjustment of tunnel orientation and can prevent an overly horizontal tunnel, surgeons must balance this advantage with the need to avoid creating an excessively steep angle. Despite these potential limitations, ongoing innovations in imaging and navigation accuracy strive to provide more precise intraoperative feedback, helping surgeons to determine an optimal, individualized tunnel trajectory that minimizes both rotational instability and tunnel enlargement risks [39,40,41]. To address these issues, innovations like enhanced imaging techniques and improved sensor technologies are continuously being developed to provide better real-time feedback and guidance to surgeons, ultimately enhancing the effectiveness of robot-assisted procedures [42]. Furthermore, excessively horizontal femoral tunnel can decrease the distance between the tunnel and the lateral femoral cortex, increasing the risk of collateral damage [16]. As demonstrated in this study, the posterior wall distance was significantly shorter in the control group compared to the robot group. The surgical robot assists surgeons in precisely designing bone tunnels with optimal angles and lengths, minimizing the risk of damage to the cartilage of the femoral medial condyle caused by an excessively short posterior wall distance.

Although primary ACL reconstruction generally demonstrates a high success rate, approximately 11–17% of patients experience graft failure within 4 years post-surgery [43]. Some studies have suggested that graft failure may be attributed to the interactions between the graft and the bone tunnel, specifically the “wiper effect” and the “bungee effect” [44,45]. The wiper effect refers to the lateral motion of the graft within the bone tunnel, while the bungee effect describes the elongation and contraction of graft along the tunnel [43]. This emphasizes the importance of the graft bending angle, as an acute graft bending angle can increase contact stress and strain at the tunnel aperture, potentially leading to graft failure [38]. In the present study, it was observed that the graft bending angle in the robot-assisted group was greater than that in the control group, indicating that the graft bending angle in the control group was more acute. An acute graft bending angle, often referred to as the “killer turn”, in transtibial reconstruction has been reported to potentially lead to graft rupture or thinning [36].

Surgical robot systems provide greater stability and precision during drilling compared to traditional handheld tools, ensuring more accurate alignment with the anatomical insertions of the ACL and enhancing surgical outcomes by maximizing graft function. These clinical advantages highlight the promising potential of robot-assisted surgery in improving surgical precision and patient outcomes. However, significant financial burdens, including the high initial purchase cost and ongoing maintenance expenses, create barriers to widespread adoption, particularly for hospitals and clinics with constrained budgets [30]. Practical challenges also arise, such as the need for specialized training for surgical staff, adjustments to workflows, and potential disruptions during the transition phase. Furthermore, disparities in access to these technologies between affluent and underserved healthcare settings raise important ethical concerns, as ensuring equitable benefits from advancements in surgical technology remains a critical issue.

The limitations of this study should be acknowledged. First, the correlation between radiographic outcomes and long-term clinical outcomes remains unclear, indicating the need for future clinical studies with extended observation periods for clinical outcomes. A second limitation is the lack of consideration of the impact of gender on the outcome data. Future studies should involve a larger sample size to allow for a more detailed analysis of outcomes based on gender. Third, this study only compared the differences in tunnel and graft characteristics between robot-assisted and traditional ACL reconstruction, without evaluating knee kinematics and function. Further research is necessary to provide additional clinical evidence to support the future application of surgical robots in anatomical ACL reconstruction. Fourth, we recognize the importance of exploring how different surgeons’ experience levels may influence results and plan to address this in future research involving a larger sample size. Additionally, the importance of integrating patient-reported outcomes, such as pain scores and functional knee scores should be acknowledged. Future research should integrate radiographic outcomes with patient-reported outcomes to provide a comprehensive evaluation of patient experiences. Lastly, despite the improved tunnel angle and graft bending angle that can be created by surgical robots, there are practical challenges to implementing robot-assisted ACL reconstruction in a clinical setting, including associated costs of robotic systems and the learning curve required for surgeons to achieve proficiency with this technology.

## 5. Conclusions

The findings of this study demonstrate that surgical robot-assisted ACL reconstruction results in significant differences in tunnel angle, graft bending angle and distance to posterior wall compared to traditional handheld locators. Specifically, the robot-assisted approach produced a larger femoral tunnel angle, a less acute graft bending angle, and a greater posterior wall distance, which are associated with enhanced safety and reduced risk of biomechanical complications such as the wiper effect and bungee effect. These results highlight the potential of surgical robots to improve the safety of ACL reconstruction, contributing to advancements in surgical practice and patient outcomes. This study provides valuable evidence supporting the clinical utility of surgical robots in improving tunnel angle and graft bending angle, which may ultimately improve postoperative knee joint stability and functional recovery.

## Figures and Tables

**Table 1:** Patient characteristics.

	Robot Group (*n* = 35)	Control Group (*n* = 35)	*p*-Value
Age, year	36.1 ± 10.2	33.4 ± 13.0	0.317
BMI, kg/m^2^	24.7 ± 4.1	25.6 ± 4.2	0.572
Sex, male/female	23/12	24/11	0.391
Time from injury to surgery, week	6.8 ± 4.3	7.4 ± 6.3	0.322
Meniscal tears, medial/lateral/both	8/2/9	4/4/9	0.793

**Table 2:** Characteristics of tunnel and graft.

	Robot Group (*n* = 35)	Control Group (*n* = 35)	*p*-Value
Femoral aperture			
Depth, %	27.9 ± 5.2	25.5 ± 9.1	0.315
Height, %	32.5 ± 4.7	30.9 ± 6.7	0.700
Tibial aperture			
Anteroposterior, %	43.0 ± 7.8	39.3 ± 9.1	0.110
Mediolateral, %	49.4 ± 2.9	48.4 ± 5.0	0.247
Femoral tunnel length, mm	35.6 ± 5.4	34.4 ± 5.4	0.386
Tibial tunnel length, mm	34.1 ± 7.5	31.5 ± 6.4	0.173
Femoral coronal angle, °	51.7 ± 7.2	46.8 ± 8.5	0.012 *
Graft bending angle, °	121.1 ± 11.7	113.4 ± 13.1	0.008 *
Posterior wall distance, mm	13.2 ± 2.9	10.3 ± 3.5	<0.001 *

* indicates statistical significance between groups (*p* < 0.05).

## Data Availability

The datasets generated and/or analyzed during the current study are not publicly available because the data are confidential patient data, but are available from the corresponding author upon reasonable request.
